# Non-equilibrium scale invariance and shortcuts to adiabaticity in a one-dimensional Bose gas

**DOI:** 10.1038/srep09820

**Published:** 2015-04-13

**Authors:** W. Rohringer, D. Fischer, F. Steiner, I. E. Mazets, J. Schmiedmayer, M. Trupke

**Affiliations:** 1 Vienna Center for Quantum Science and Technology, Atominstitut, TU Wien, 1020 Vienna, Austria; 2 Ioffe Physical-Technical Institute of the Russian Academy of Sciences, 194021 St. Petersburg, Russia; 3 Wolfgang Pauli Institute, 1090 Vienna, Austria

## Abstract

We present experimental evidence for scale invariant behaviour of the excitation spectrum in phase-fluctuating quasi-1d Bose gases after a rapid change of the external trapping potential. Probing density correlations in free expansion, we find that the temperature of an initial thermal state scales with the spatial extension of the cloud as predicted by a model based on adiabatic rescaling of initial eigenmodes with conserved quasiparticle occupation numbers. Based on this result, we demonstrate that shortcuts to adiabaticity for the rapid expansion or compression of the gas do not induce additional heating.

A systematic understanding of non-equilibrium dynamics in many-body quantum systems is a longstanding goal, with far-reaching applicability for many different fields of physics. Ultracold atom experiments offer clean implementations of systems that are tunable, well isolated from the environment and theoretically tractable^1^,^2^. In particular, the profound understanding available for the one-dimensional (1d) Bose gas makes it an ideal test bed for quantum many-body dynamics^3^.

Tunable parameters in the system's Hamiltonian allow the controlled preparation of non-equilibrium states^4^,^5^,^6^,^7^. The identification of characteristic scaling laws is an important step for the concise description of the subsequent dynamical processes. Of particular importance are laws governing not only global parameters^8^,^9^,^10^ but ideally the full spectrum of excitations, as studied in recent experiments with 2d Bose^11^,^12^ or Tonks-Girardeau gases^4^,^13^.

Recent work^14^ has shown that a general scaling property of many-body wavefunctions holds exactly for a broad class of systems, including the weakly interacting 1d Bose gas addressed in this Letter. The existence of such a scaling solution is a consequence of a dynamical symmetry of the underlying Hamiltonian. For an ultracold gas, fast changes of control parameters in the Hamiltonian generally lead to quasiparticle production and heating^15^. The existence of a scaling solution for the full spectrum of quasiparticle modes implies that so-called shortcuts to adiabaticity (STA)^16^,^17^ can be engineered not only for the mean density profile of a 1d gas, but also for correlation properties of the system in certain regimes of interaction strength^18^,^19^.

We show in this work that the scaling solutions for a true many-body wavefunction have their counterpart in the hydrodynamic regime of our experimental system. We bring our system out of equilibrium by rapidly changing its longitudinal confinement. The subsequent system evolution gives insight into the scaling properties of the gas. This allows us to study the regimes and limits of such a manipulation, with an emphasis on STA schemes. We furthermore demonstrate for the first time that STA schemes are valid for the second-order correlation, and thereby the temperature, of weakly interacting 1d Bose gases.

## Results and Discussion

In our experiments, we investigate the scaling solutions of hydrodynamic equations and how they can be applied for the rapid control of the complete wavefunction of a many-body quantum system.

We start with a single quasicondensate of several thousand ^87^Rb atoms in an elongated trap on an atom chip^20^. The initial temperatures are set between 50 nK and 150 nK and linear densities range between 50 atoms/*μ*m and 200 atoms/*μ*m. Axially, the cloud is deeply in the Thomas-Fermi regime. Radially, the gas is described by an interaction-broadened ground state wavefunction^21^,^22^,^23^. For these parameters, both the chemical potential and the average thermal energy per particle fulfil the condition 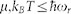, where 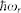 denotes the radial level spacing of the trap with frequency 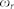, so that scattering into radial excited states is strongly suppressed and an effective 1d system is realized^24^,^20^,^25^,^21^. After evaporative cooling, we keep an RF-shield 12 kHz above the trap bottom throughout our experiments to remove hot atoms. The cloud is probed by standard absorption imaging techniques after a 4 ms to 10 ms long phase of time-of-flight expansion.

**Figure 1:**
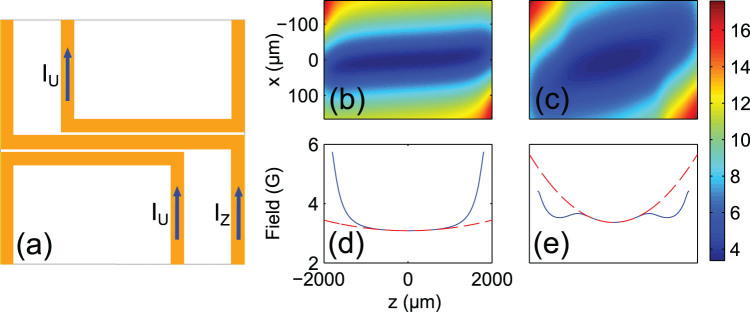
Time-dependent potentials on an atom chip. (a) The current ratio between a central Z-shaped wire and two U-shaped control wires allows us to precisely tune the trap geometry. For a symmetric current flow, the trap minimum is positioned below the center of the Z-wire, with the long trap axis aligned to the horizontal direction. (b) 2d cut through the trapping potentials for 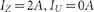 and (c) 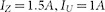 at a constant external Bias field of 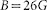, respectively. (d,e) Cuts through the radial trap minimum of the same potentials to show the axial trap deformation.

The geometry of the trap is governed by the current flow through a central Z-shaped wire and two U-shaped control structures on the atom chip, as shown in figure 1(a). Panels (b)–(e) show two different trapping potentials calculated for currents tuned to 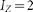 A and 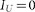 A, as well as 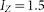 A and 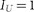 A. Varying 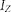 and 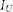 results in traps with axial confinement ranging from 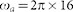 Hz to 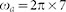 Hz, and radial confinement from 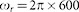 Hz to 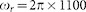 Hz. A rapid change of the current ratio 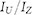 constitutes a quench of the trapping potential and induces excitations.

In our first set of experiments we probe the dynamical scaling of the phonon ensemble in the presence of an axial quadrupole-mode collective excitation^26^ induced by such a quench. To this end, we employ a linear ramp from 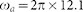 Hz to 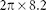 Hz, and from 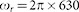 Hz to 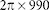 Hz, respectively, of duration *τ*. The ramps of the trapping potential were designed to avoid transverse excitations. We chose to maintain a constant transverse position to avoid inducing a corresponding sloshing of the cloud. The ramp duration was chosen to be longer than 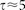 ms so that adiabaticity with respect to the change of transverse trap frequency is fulfilled. Axial dipole oscillations are suppressed by the symmetric arrangement of the control wires.

**Figure 2:**
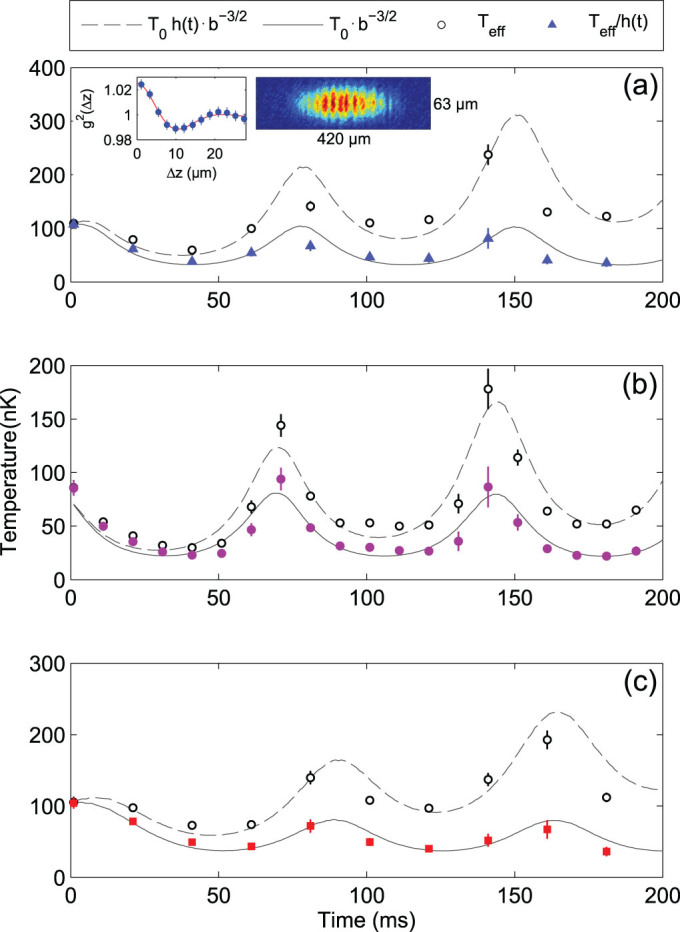
Temperature evolution following a quench. Black circles: temperatures measured from density correlations in free expansion. Dashed lines: scaling law taking into account heating as described by the expression 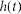 discussed in the methods section, fitted for effective rates for each dataset. Blue triangles, purple circles and red squares:temperatures corrected for heating rate. Lines: scaling law 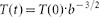 as discussed in the main text. Error bars represent the standard error estimated by a bootstrapping technique, as used in ref. 30. (a) Quench time *τ* = 10 ms, atom number 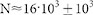, heating rate 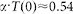 nK/ms. Inset: thermometry with density correlations in free expansion. Data points correspond to an average of autocorrelations over 350 density profiles integrated from pictures as depicted here. (b) *τ* = 10 ms, 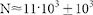, heating rate 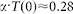 nK/ms. (c) 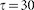 ms, 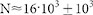, 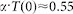 nK/ms.

We probe phononic excitations in the quasicondensate using a thermometry scheme based on the analysis of density correlations in free expansion^27^,^28^, as shown in the inset of figure 2(a). To extract the temperature we compare the measured density correlation functions with the results of a stochastic model^29^. Our analysis accounts for the effects of the collective excitation on the free expansion (see methods section below), and for the finite resolution of our imaging system.

Figure 2 summarises our temperature measurements following a quench. We show data for ramp times of 10 and 30 ms and mean atom numbers of 11000 and 16000, compared to the behaviour expected from a scaling model building upon the results of Ref^14^.

The scale invariance of the underlying Hamiltonian allows to calculate time-dependent correlation functions: In the Thomas-Fermi regime, the density profile exhibits self-similar scaling described by
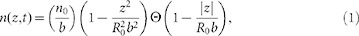
with a time-dependent scale factor 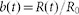. Here, 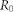 and 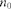 denote the initial Thomas-Fermi radius and peak density, respectively, 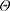 is the Heaviside function and *z* represents the axial coordinate. The scale factor obeys an Ermakov-like equation^31^
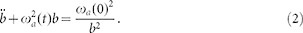
Using the rescaled mean-field density (1), we can write the linearised hydrodynamic equations for density and velocity fluctuations 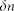 and 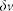, disregarding the quantum pressure term, as
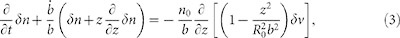
and
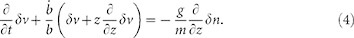
To solve these equations we introduce an ansatz of rescaled eigenmodes for density and phase fluctuations. This approach yields a set of uncoupled equations and hence no mixing of modes, finally predicting an adiabatic time evolution of the corresponding occupation numbers. For a thermal state, the initial phonon occupation numbers are given by a Bose distribution
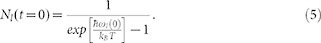
Adiabaticity results in a constant ratio 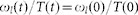. The spectrum at 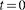 is given by^32^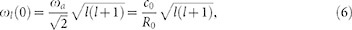 with mode index *l* and initial sound velocity 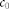. For 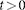, it scales as 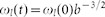, due to the time-dependence of the sound velocity 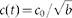 and radius 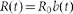. Hence, for an initial state in thermal equilibrium, we obtain the temperature scaling
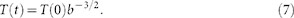
The density correlations in free expansion that our thermometry scheme relies on are governed by the coherence function. For a thermal state with homogeneous density, as realised in the vicinity of the cloud center, it has the form^20^,^32^:
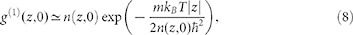
where 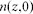 denotes the density at time 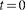 and 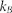 the Boltzmann constant. Based on our model, the coherence function is expected to scale as
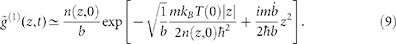


**Figure 3:**
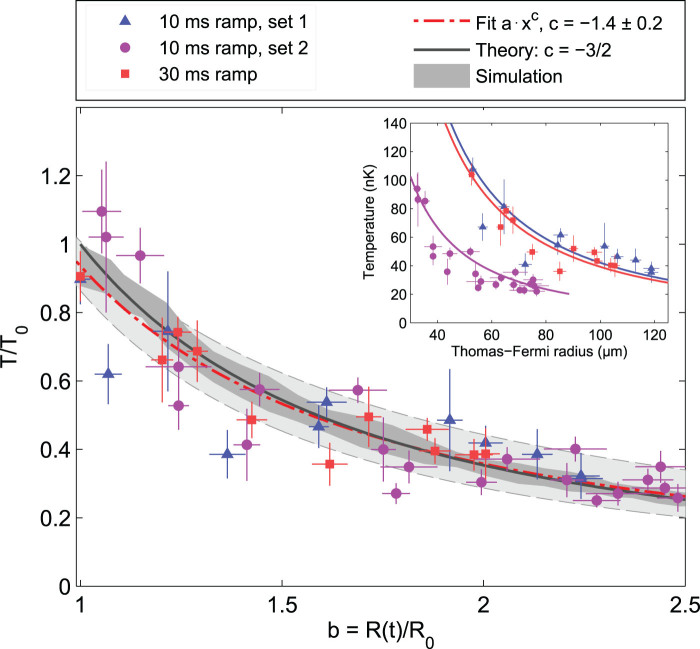
Temperature as a power law of the scaling factor. Main figure: datasets presented in figure 2(a) (blue triangles), 2(b) (purple circles) and 2(c) (red squares), respectively, recast in units of the initial temperature as a function of the scaling factor. Inset: data in absolute units. Vertical error bars are standard errors resulting from a bootstrapping method as applied in ref. 30. Horizontal error bars correspond to the error of measured cloud widths, normalised to the initial width. Dash-dotted line: power law fit to the data, with the lightly shaded area representing the fit's 95% confidence bounds. Black line: scaling model. Dark shaded area: classical field simulation with 120 sets of stochastic initial conditions generated by a SGPE. The plotted data is corrected for the independently measured heating rate.

Figure 3 summarizes the first central result of our experiments: The inset shows absolute temperatures plotted against measured Thomas-Fermi radii. If the measured temperatures are scaled to the initial temperature and plotted against the scale parameter 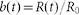, the datasets collapse onto a single line. This illustrates a scaling behaviour that is universal in sense that it is independent of absolute temperature, density or quench time. To validate our results we furthermore performed numerical simulations based on a stochastic Gross-Pitaevskii equation (SGPE)^33^,^34^,^35^,^36^, showing excellent agreement with the scaling model (fig. 3).

So far, we considered the dynamics induced by a linear ramp of the trapping potential. In the following, we demonstrate the conservation of phonon occupation numbers during shortcuts to adiabaticity^31^,^18^,^17^ for the rapid expansion and compression of a 1d quasi-BEC. To implement these shortcuts, we make use of an optimal control approach that is in spirit similar to the method proposed in ref. 37. We numerically solve the time-dependent 1d GPE with a suitable parametrisation of the trap which is subject to a global optimization procedure based on a genetic algorithm^38^,^39^. The ramp speed is limited by the requirement of adiabaticity in the transverse degree of freedom. This constraint also guarantees that the gas remains in the 1d hydrodynamic regime, and that the interaction strength varies slowly with time. The properties of the ultracold gas therefore remain consistent with the conditions necessary for the validity of the microscopic scaling laws^14^ throughout the ramp.

**Figure 4:**
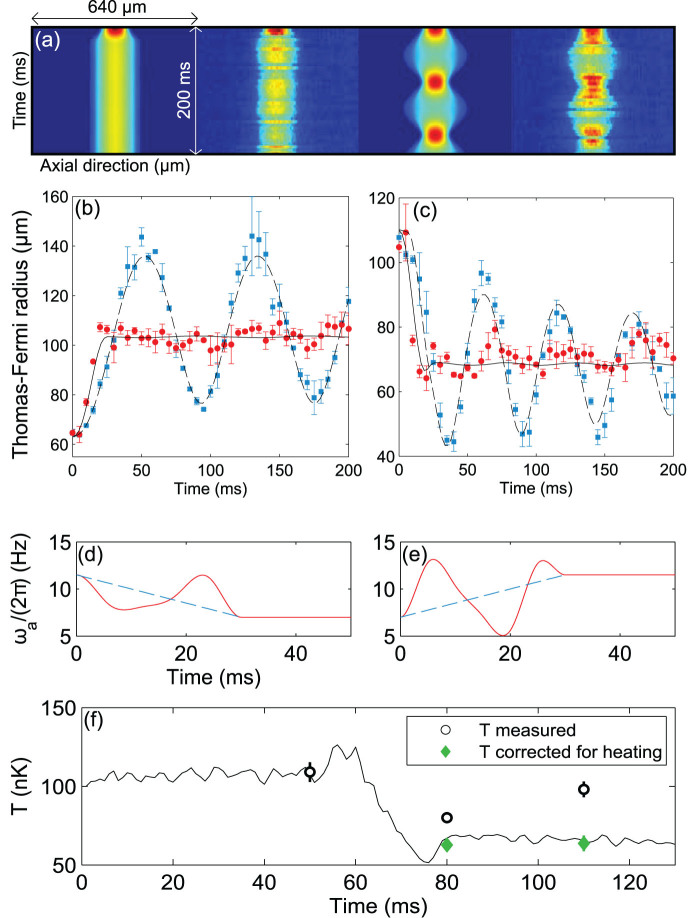
STA for fast confinement changes. (a) Density profiles for optimal and linear ramps in simulation and experiment. Experimental profiles are averaged of 5 shots at identical parameters, taken at a free expansion time of 5 ms. (b) Measured Thomas-Fermi radii for an optimal decompression (red circles) from 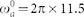 Hz, 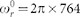 Hz to 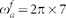 Hz, 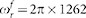 Hz, and for a linear ramp (blue squares) compared with results from a GPE simulation (black and black-dashed lines). (c) Measured Thomas-Fermi radii after an optimal (red circles), and a linear ramp (blue squares) for a compression of the cloud, inverting initial and final trap frequencies as given for panel (b), again compared with GPE simulation results including damping (black and black-dashed lines). (d),(e) Optimal trap frequency ramp for decompression (d) and compression (e) within 30 ms (red line). Dashed lines: corresponding linear ramps. (f) Temperature measurements before and after the STA with (green diamonds) and without correction for extrinsic heating rate (black circles), compared to simulation results (black line).

The upper panel in figure 4 shows a comparison between simulation and experiment for a linear and a shortcut ramp performing a decompression within 30 ms from a trap with frequencies 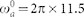 Hz and 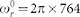 Hz to 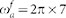 Hz and 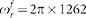 Hz. The subsequent dynamics is observed throughout a period of 170 ms, each picture taken after a short free expansion time of 5 ms, showing excellent agreement with simulations. It is interesting to note that our shortcut ramps are similar to theoretical results derived from a counter-diabatic driving method reported recently^19^.

For the STA, we expect an adiabatic state change, defined by 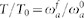. The temperature measurements, corrected for the measured heating rate, are in good agreement with the adiabatic prediction of 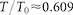 for the implemented decompression shortcut, confirming that there is no additional heating during the applied procedure.

## Conclusion

In summary, we have characterised the temperature of the phonon ensemble in a breathing quasi-1d Bose gas for different initial conditions, and used it to test the predicted dynamical scale invariance in the excitation spectrum of a quasi-1d Bose gas. Following these scaling laws, we have experimentally demonstrated rapid adiabatic expansion and compression of a 1d Bose gas in the hydrodynamic regime, allowing fast transformation of the trapped cloud without additional heating.

Our work is only the beginning for studies of many-body scaling solutions and shortcuts to adiabaticity. The existence of scaling solutions has been proposed for a large class of cold atom systems^14^. In principle, this opens up the interesting possibility to apply the techniques applied here to a variety of settings, such as fermionic systems or the 1d Bose gas with intermediate or strong interactions. We expect that studying the effect of quasiparticle interactions on the implementation of shortcuts to adiabaticity will shed new light on the complex many-body dynamics in these systems, in addition to providing novel tools for their controlled manipulation.

We expect that such extensions to studies in regimes of greater interaction strength, and to systems out of thermal equilibrium, will benefit from the tools presented in this work.

## Methods

### Condensate preparation and detection

We employ standard cooling and magnetic trapping techniques^40^ to prepare ultracold quasi-one-dimensional samples of 87Rubidium atoms in the 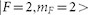 state on an atom chip^20^,^41^. Atom chips feature microfabricated wire structures to create fields for atom trapping and manipulation^42^. The structures used in our experiments are produced by masked vapor depositon of a 2 *μm* gold layer on a silicon substrate, with a width of both trapping and control wires of 200 *μm*. For detection, we employ resonant absorption imaging^43^ using a high quantum-efficiency CCD camera (Andor iKon-M 934 BR-DD) and a diffraction-limited optical imaging system characterised by an Airy radius of 4.5 *μm*. The RF shield at 12 kHz above the bottom of the trap is used to limit the number of atoms in the thermal background cloud populating transverse excited states of the trap, which would otherwise adversely affect our thermometry scheme by reduction of interference contrast in free expansion.

### Characterization of the breathing mode

**Figure 5:**
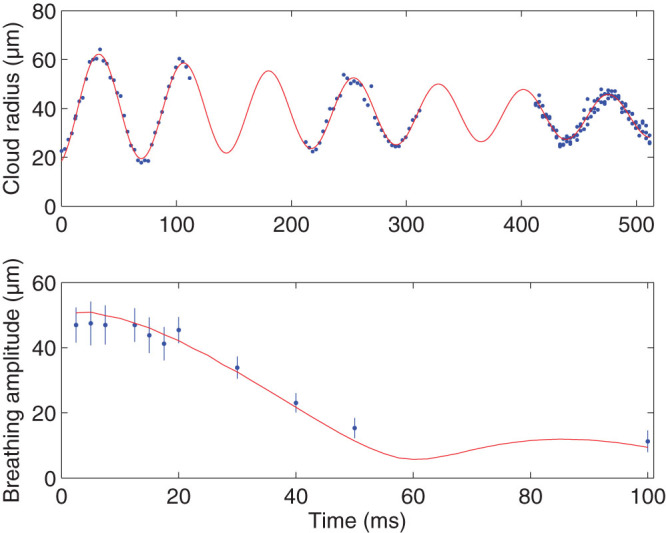
Characterization of the breathing mode induced by a trap quench. Upper panel: Breathing induced by a linear quench during a time 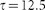 ms. The fit includes an exponential damping term, with a time constant 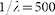 ms. Lower panel: Breathing amplitude plotted against quench time *τ*. Error bars correspond to 95% confidence intervals of fits as shown in the upper panel. The theoretical calculations (line) are based on numerically solving a 1d GPE.

We characterise the breathing mode excited by a linear trap frequency ramp from 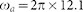 Hz to 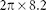 Hz, and 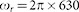 Hz to 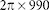 Hz, respectively, in figure 5. As an example, the upper panel shows the time evolution of the cloud radius after a ramp with duration *τ* = 12.5 ms. Fitting data as presented here allows us to extract frequencies, damping rates and amplitudes of the breathing mode. The frequency 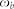 is influenced by the total atom number in the trap, and is expected to vary with the axial trap frequency between 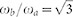 in the 1d limit, and 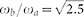 representing the elongated 3d regime^26^. The amplitude strongly depends on the duration and shape of the trap frequency ramp. The lower panel in figure 5 shows a comparison of measured breathing amplitudes for different ramp times between 2 ms and 100 ms with results calculated with a 1d Gross-Pitaevskii equation (GPE), taking into account corrections to the interaction term relevant in the 1d/3d crossover regime^44^, and shows good agreement in the chosen parameter range.

### Thermometry

In this work we use the thermometry scheme proposed and demonstrated in ref. 28,27 based on the analysis of density correlations in freely expanding phase-fluctuating quasi-1d condensates and comparison with numerically calculated density profiles^29^.

Breathing contributes a velocity field characterized by the derivative of the scale parameter 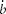, leading to an additional axial compression or expansion of the density profile during free expansion. This effect can be accounted for by an additional phase factor
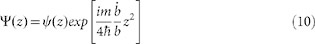
in the numerics, where *b* and 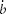 are determined by fits to the measured breathing oscillations. The error on the temperature measurements is estimated by a bootstrapping method as outlined in ref. 30.

### Derivation of the temperature scaling

The general conditions for the existence of a scaling solution are stated in reference^14^. For the 1d Bose gas, they are fulfilled in the presence of contact interactions, as well as a harmonic, linear or vanishing axial trapping potential. Given that our system is a 1d quasicondensate, and the trapping potential is harmonic, we can derive the corresponding hydrodynamic scaling relations for correlation functions. Our starting point is the self-similar scaling of the density profile:
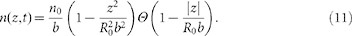

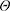
 denotes the Heaviside function, 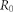 the initial Thomas-Fermi radius and *b* the scale parameter. Similar to the discussion of the corresponding equilibrium problem^32^, a scaling solution in terms of eigenmodes for density and velocity fluctuations 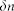 and 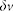 can be formulated as
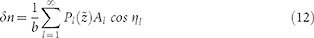
and
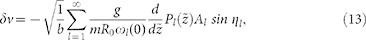
with the Legendre polynomials 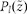, the interaction constant *g*, rescaled coordinates 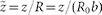, and time-dependent amplitudes 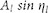 and 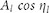. 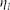 denotes the frequency of the oscillation between the quadratures of the mode *l*. Correspondingly, the initial equilibrium spectrum scales as
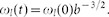
Substituting 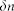 and 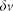 into the linearised Euler equations
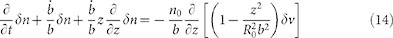

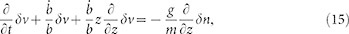
where we have disregarded the quantum pressure term, yields
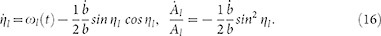
Since the characteristic inverse time scale of the breathing mode 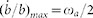 is small compared to the characteristic frequencies 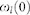 of the phonon modes with 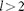, we can average over rapid oscillations of 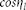 and 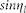 to reduce these expressions to 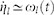 and 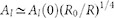, and the phonon modes are expected to scale adiabatically. Then the initial number of phonons in a thermal state,
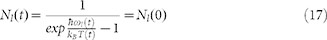
is conserved, resulting in
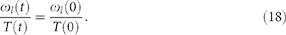
This leads to the observed temperature scaling 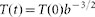.

The decay of the coherence function of a quasicondensate is dominated by phase noise^45^. We can express phase fluctuations in terms of velocity fluctuations using the relation
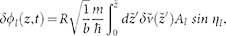
where
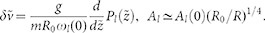


Therefore the relation between initial and time-dependent modes 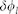 reads
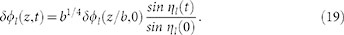
The time-dependent one-body reduced density matrix can be expressed as
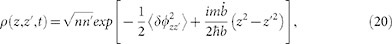
with
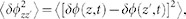
as well as 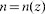 and 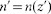. Using 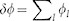, we can write the density matrix (20) in terms of the modes given in equation (19). Substituting and following the steps in reference^45^, we find that near the cloud center, where the density is practically uniform and we can use trigonometric approximations for 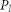
46,
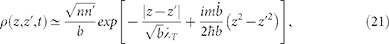
with a coherence length 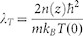. This corresponds to a transformation of the form
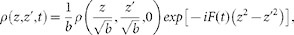
as predicted in reference^14^, with the difference that the spatial coordinates scale with 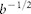 instead of 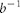. This difference is a consequence of the the Thomas-Fermi approximation. In the hydrodynamic regime, scale invariance therefore holds even if the interaction strength is kept constant. In contrast, reference^14^ assumes a suitable tuning of the interaction constant, thereby yielding an exact solution valid for arbitrary values of the Lieb-Liniger parameter.

### Heating

The temperature scaling 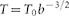 satisfies the equation
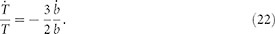
In our experiment we observe heating during evolution times of several hundreds of milliseconds. We find that all our measurements are compatible with a linear increase of temperature over time, which can be represented by adding a constant heating term to the equation:
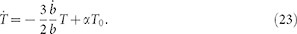
This equation is solved by 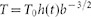, with 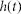 given by

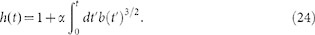
The integral can be calculated numerically and *α* corresponds to the regular heating rate in units of the initial temperature for constant *b*.

### Finite temperature simulations

We solve a stochastic 1d Gross-Pitaevskii equation (SGPE)^33^,^34^,^35^,^36^ of the form
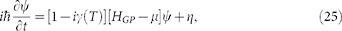
where
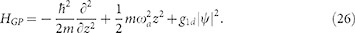
Here *μ* denotes an external chemical potential, and 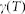 is a damping coefficient that is coupled to the *δ*-correlated noise term *η* via a fluctuation-dissipation theorem:
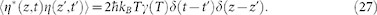
Repeated solution of the SGPE yields a set of independent wave functions representing a thermal state. We use this state as initial condition for propagation with a time-dependent Gross-Pitaevskii Hamiltonian without any noise or damping terms. Such an approach has previously been applied to model condensate formation in atom chip traps^47^ and is very similar to other classical field methods based on stochastic sampling of initial conditions^48^,^49^. The simulation results are analysed with the same procedures as the experimental data.

## Author Contributions

W.R. and D.F. performed the experiments and analysed the data. F.S. contributed to building the experiment and provided help with the data analysis. I.E.M. provided important advice for the execution of the scaling measurements and developed the theoretical model. W.R. devised the optimal control scheme and performed numerical simulations. J.S. and M.T. provided scientific guidance and funding for the experiment. All authors contributed to the interpretation of the data and the writing of the manuscript.

## References

[b1] Polkovnikov A., Sengupta K., Silva A. & Vengalattore M. Colloquium: Nonequilibrium dynamics of closed interacting quantum systems. Rev. Mod. Phys. 83, 863–883 (2011).

[b2] Rigol M., Dunjko V. & Olshanii M. Thermalization and its mechanism for generic isolated quantum systems. Nature 452, 854–8 (2008). 18421349 10.1038/nature06838

[b3] Cazalilla M. A., Citro R., Giamarchi T., Orignac E. & Rigol M. One dimensional bosons: From condensed matter systems to ultracold gases. Rev. Mod. Phys. 83, 1405–1466 (2011).

[b4] Kinoshita T., Wenger T. & Weiss D. S. A quantum Newton's cradle. Nature 440, 900–3 (2006). 16612376 10.1038/nature04693

[b5] Sadler L. E., Higbie J. M., Leslie S. R., Vengalattore M. & Stamper-Kurn D. M. Spontaneous symmetry breaking in a quenched ferromagnetic spinor Bose-Einstein condensate. Nature 443, 312–5 (2006). 16988706 10.1038/nature05094

[b6] Cheneau M. *et al.* Light-cone-like spreading of correlations in a quantum many-body system. Nature 481, 484–487 (2012). 22281597 10.1038/nature10748

[b7] Gring M. *et al.* Relaxation and prethermalization in an isolated quantum system. Science 337, 1318–1322 (2012). 22956685 10.1126/science.1224953

[b8] Kagan Y., Surkov E. L. & Shlyapnikov G. V. Evolution of a Bose-condensed gas under variations of the confining potential. Phys. Rev. A 54, R1753–R1756 (1996). 9913756 10.1103/physreva.54.r1753

[b9] Castin Y. & Dum R. Bose-Einstein Condensates in Time Dependent Traps. Phys. Rev. Lett. 77, 5315–5319 (1996). 10062773 10.1103/PhysRevLett.77.5315

[b10] Chevy F., Bretin V., Rosenbusch P., Madison K. W. & Dalibard J. Transverse Breathing Mode of an Elongated Bose-Einstein Condensate. Phys. Rev. Lett. 88, 250402 (2002). 12097077 10.1103/PhysRevLett.88.250402

[b11] Pitaevskii L. P. & Rosch A. Breathing modes and hidden symmetry of trapped atoms in two dimensions. Phys. Rev. A 55, 853–856 (1997).

[b12] Hung C.. -L. Zhang, X., Gemelke, N. & Chin, C. Observation of scale invariance and universality in two-dimensional Bose gases. Nature 470, 236–9 (2011). 21270797 10.1038/nature09722

[b13] Minguzzi A. & Gangardt D. M. Exact Coherent States of a Harmonically Confined Tonks-Girardeau Gas. Phys. Rev. Lett. 94, 240404 (2005).

[b14] Gritsev V., Barmettler P. & Demler E. Scaling approach to quantum non-equilibrium dynamics of many-body systems. New J. Phys. 12, 113005 (2010).

[b15] Fedichev P. & Fischer U. Cosmological quasiparticle production in harmonically trapped superfluid gases. Phys. Rev. A 69, 033602 (2004).

[b16] Chen X., Ruschhaupt A., Schmidt S. & Muga J. G. Shortcut to adiabaticity in harmonic traps. J. At. Mol. Sci. 1, 1–17 (2010). 10.1103/PhysRevLett.104.063002 20366818

[b17] Schaff J.. -F.., Capuzzi, P., Labeyrie, G. & Vignolo, P. Shortcuts to adiabaticity for trapped ultracold gases. New J. Phys. 13, 113017 (2011).

[b18] del Campo A. Frictionless quantum quenches in ultracold gases: A quantum-dynamical microscope. Phys. Rev. A 84, 4–7 (2011).

[b19] del Campo A. Shortcuts to Adiabaticity by Counterdiabatic Driving. Phys. Rev. Lett. 111, 100502 (2013). 25166641 10.1103/PhysRevLett.111.100502

[b20] Reichel J. & Vuletic V. Atom Chips. Atom Chips (Wiley, 2010).

[b21] Salasnich L., Parola A. & Reatto L. Effective wave equations for the dynamics of cigar-shaped and disk-shaped Bose condensates. Phys. Rev. A 65, 43614 (2002).

[b22] Krüger P., Hofferberth S., Mazets I. E., Lesanovsky I. & Schmiedmayer J. Weakly Interacting Bose Gas in the One-Dimensional Limit. Phys. Rev. Lett. 105, 265302 (2010). 21231675 10.1103/PhysRevLett.105.265302

[b23] Amerongen A. H. V., Es J. J. P. V., Wicke P., Kheruntsyan K. V. & Druten N. J. V. Yang-Yang Thermodynamics on an Atom Chip. Phys. Rev. Lett. 090402, 13–15 (2008). 10.1103/PhysRevLett.100.090402 18352680

[b24] Görlitz A. *et al.* Realization of Bose-Einstein Condensates in Lower Dimensions. Phys. Rev. Lett. 87, 130402 (2001). 11580572 10.1103/PhysRevLett.87.130402

[b25] Stringari S. Dynamics of Bose-Einstein condensed gases in highly deformed traps. Phys. Rev. A 58, 2385–2388 (1998).

[b26] Menotti C. & Stringari S. Collective oscillations of a one-dimensional trapped Bose-Einstein gas. Phys. Rev. A 66, 043610 (2002).

[b27] Manz S. *et al.* Two-point density correlations of quasicondensates in free expansion. Phys. Rev. A 81, 1–4 (2010).

[b28] Imambekov A. *et al.* Density ripples in expanding low-dimensional gases as a probe of correlations. Phys. Rev. A 80, 1–14 (2009).

[b29] Stimming H.. -F.., Mauser, N. J., Schmiedmayer, J. & Mazets, I. E. Fluctuations and Stochastic Processes in One-Dimensional Many-Body Quantum Systems. Phys. Rev. Lett. 105, 015301 (2010). 20867458 10.1103/PhysRevLett.105.015301

[b30] Kuhnert M. *et al.* Multimode Dynamics and Emergence of a Characteristic Length Scale in a One-Dimensional Quantum System. Phys. Rev. Lett. 110, 090405 (2013). 23496695 10.1103/PhysRevLett.110.090405

[b31] Chen X., Lizuain I., Ruschhaupt A., Guéry-Odelin D. & Muga J. G. Shortcut to Adiabatic Passage in Two- and Three-Level Atoms. Phys. Rev. Lett. 105, 123003 (2010). 20867634 10.1103/PhysRevLett.105.123003

[b32] Petrov D., Shlyapnikov G. & Walraven J. T. M. Regimes of Quantum Degeneracy in Trapped 1D Gases. Phys. Rev. Lett. 85, 3745–3749 (2000). 11041917 10.1103/PhysRevLett.85.3745

[b33] Stoof H. T. C. Coherent Versus Incoherent Dynamics During Bose-Einstein Condensation in Atomic Gases. J. Low Temp. Phys. 114, 11–109 (1999).

[b34] Duine R. & Stoof H. Stochastic dynamics of a trapped Bose-Einstein condensate. Phys. Rev. A 65, 013603 (2001).

[b35] Gardiner C., Anglin J. & Fudge T. The stochastic Gross-Pitaevskii equation. J. Phys. B At. Mol. Opt. Phys. 35, 1555–1582 (2002).

[b36] Cockburn S. P., Gallucci D. & Proukakis N. P. Quantitative study of quasi-one-dimensional Bose gas experiments via the stochastic Gross-Pitaevskii equation. Phys. Rev. A 84, 023613 (2011).

[b37] Caneva T., Calarco T. & Montangero S. Chopped random-basis quantum optimization. Phys. Rev. A 84, 022326 (2011).

[b38] Holland J. H. Adaptation in Natural and Artificial Systems: An Introductory Analysis with Applications to Biology, Control, and Artificial Intelligence. A Bradford book (M.I.T.P., 1992).

[b39] Rohringer W. *et al.* Stochastic optimization of a cold atom experiment using a genetic algorithm. Appl. Phys. Lett. 93, 264101 (2008).

[b40] Wildermuth S. *et al.* Optimized magneto-optical trap for experiments with ultracold atoms near surfaces. Phys. Rev. A 69, 030901 (2004).

[b41] Folman R. *et al.* Controlling cold atoms using nanofabricated surfaces: atom chips. Phys. Rev. Lett. 84, 4749–52 (2000). 10990787 10.1103/PhysRevLett.84.4749

[b42] Groth S. *et al.* Atom chips: Fabrication and thermal properties. Appl. Phys. Lett. 85, 2980 (2004).

[b43] Smith D. A. *et al.* Absorption imaging of ultracold atoms on atom chips. Opt. Express 19, 8471–85 (2011). 21643097 10.1364/OE.19.008471

[b44] Gerbier F. Quasi-1D Bose-Einstein condensates in the dimensional crossover regime. Europhys. Lett. 66, 771–777 (2004).

[b45] Mora C. & Castin Y. Extension of Bogoliubov theory to quasicondensates. Phys. Rev. A 67, 053615 (2003).

[b46] Abramowitz M. & Stegun I. A. Handbook of mathematical functions with formulas, graphs, and mathematical tables, vol. 55 of *National Bureau of Standards Applied Mathematics Series* (For sale by the Superintendent of Documents, U.S. Government Printing OfficeWashington, D.C., 1964).

[b47] Proukakis N., Schmiedmayer J. & Stoof H. Quasicondensate growth on an atom chip. Phys. Rev. A 73, 053603 (2006).

[b48] Sinatra A., Lobo C. & Castin Y. The truncated Wigner method for Bose-condensed gases: limits of validity and applications. J. Phys. B At. Mol. Opt. Phys. 35, 3599–3631 (2002).

[b49] Witkowska E., Gajda M. & Rzazewski K. Monte Carlo method, classical fields and Bose statistics. Opt. Commun. 283, 671–675 (2010).

